# Molecular Phylogenetic Diversity and Biological Characterization of *Diaporthe* Species Associated with Leaf Spots of *Camellia sinensis* in Taiwan

**DOI:** 10.3390/plants10071434

**Published:** 2021-07-14

**Authors:** Hiran A. Ariyawansa, Ichen Tsai, Jian-Yuan Wang, Patchareeya Withee, Medsaii Tanjira, Shiou-Ruei Lin, Nakarin Suwannarach, Jaturong Kumla, Abdallah M. Elgorban, Ratchadawan Cheewangkoon

**Affiliations:** 1 Department of Plant Pathology and Microbiology, College of Bioresources and Agriculture, National Taiwan University, Taipei 10617, Taiwan; ichenntsaii@gmail.com (I.T.); r07633020@gmail.com (J.-Y.W.); 2 Biodiversity and Climate Research Centre (BiK-F), 60325 Frankfurt am Main, Germany; 3 Department of Biological Science, Goethe University Frankfurt, 60438 Frankfurt am Main, Germany; 4 Department of Entomology and Plant Pathology, Faculty of Agriculture, Chiang Mai University, Chiang Mai 50200, Thailand; pwithee92@gmail.com (P.W.); t_medsaii@hotmail.com (M.T.); 5 Department of Tea Agronomy, Tea Research and Extension Station, Taoyuan 32654, Taiwan; tres226@ttes.gov.tw; 6 Research Centre of Microbial Diversity and Sustainable Utilization, Faculty of Science, Chiang Mai University, Chiang Mai 50200, Thailand; suwan.462@gmail.com (N.S.); jaturong_yai@hotmail.com (J.K.); 7 Innovative Agriculture Research Center, Faculty of Agriculture, Chiang Mai University, Chiang Mai 50200, Thailand; 8 Department of Botany and Microbiology, College of Science, King Saud University, Riyadh 11451, Saudi Arabia; aelgorban@ksu.edu.sa

**Keywords:** endophytes, foliar pathogens, pathogenicity, taxonomy

## Abstract

*Camellia sinensis* is one of the major crops grown in Taiwan and has been widely cultivated around the island. Tea leaves are prone to various fungal infections, and leaf spot is considered one of the major diseases in Taiwan tea fields. As part of a survey on fungal species causing leaf spots on tea leaves in Taiwan, 19 fungal strains morphologically similar to the genus *Diaporthe* were collected. ITS (internal transcribed spacer), *tef1-α* (translation elongation factor 1-α), *tub2* (beta-tubulin), and *cal* (calmodulin) gene regions were used to construct phylogenetic trees and determine the evolutionary relationships among the collected strains. In total, six *Diaporthe* species, including one new species, *Diaporthe hsinchuensis*, were identified as linked with leaf spot of *C. sinensis* in Taiwan based on both phenotypic characters and phylogeny. These species were further characterized in terms of their pathogenicity, temperature, and pH requirements under laboratory conditions. *Diaporthe tulliensis*, *D. passiflorae*, and *D. perseae* were isolated from *C. sinensis* for the first time. Furthermore, pathogenicity tests revealed that, with wound inoculation, only *D. hongkongensis* was pathogenic on tea leaves. This investigation delivers the first assessment of *Diaporthe* taxa related to leaf spots on tea in Taiwan.

## 1. Introduction

Species of *Diaporthe* have been frequently reported as pathogens, endophytes, and saprobes of various types of hosts [1,2,3,4,5,6,7,8,9,10,11,12,13,14,15,16,17]. These taxa have been reported globally and cause various diseases on economically important plants and crops such as dieback of forest trees [2], leaf spots on tea and *Ixora* spp. [3,4], leaf and pod blights and seed decay in soybean [5], melanose and stem-end rot on *Citrus* spp. [6,7,8,9,10], leaf spot on common hop [11], twig blight and dieback of blueberry [12], trunk diseases of grapevines [13], branch canker on *Cinnamomum camphora* (Camphor Tree) [14], pear shoot canker [15], and stem canker on sunflower [16,17].

The genus *Diaporthe (*syn. *Phomopsis)* was introduced by Nitschke and is typified by *D. eres* [18,19,20,21,22,23,24,25,26,27]. It is categorized in the family Diaporthaceae and order Diaporthales [18,19,20,21,22,23,24,25,26,27]. The holomorphic name for *Diaporth*e/*Phomopsis* was complex. Therefore, following the nomenclature rules, Rossman et al. [28] recommended adopting the older sexual typified name *Diaporthe* over the younger asexual typified name *Phomopsis*. Currently, Index Fungorum (retrieved in April 2021) reported over 900 names under the genus *Phomopsis,* whereas *Diaporthe* contains over 1000 names. Species of *Diaporthe* were traditionally identified based on their phenotypic characters such as colony morphology, appearance of ascomata and conidiomata, variation in ascospores and conidiospores, and host affiliations [18,19,20,21,22,23,24,25,26,27]. However, due to improvements in DNA sequencing, various enigmatic taxa have been discovered, which has reformed our understanding of the natural classification of *Diaporthe*. Recent studies based on molecular phylogeny have continuously discovered that traditionally used morphological characters and host associations are not sufficient to determine the species of *Diaporthe* strains because they show variation under different environmental conditions [29]. Therefore, multilocus phylogenies based on DNA sequences of ITS (internal transcribed spacer), *tef1-α* (translation elongation factor 1-α), *tub*2 (beta-tubulin), and *cal* (calmodulin) are more often used to determine the natural classification of *Diaporthe* species [1,26,29,30,31]. 

Foliar fungal pathogens that infect *Camellia sinensis* can lead to a notable reduction in their yield, resulting in a loss of income [3,32,33]. Tea leaves at all stages are susceptible to fungal diseases [3,32,33]. In total, 520 fungal species have been identified to occur on *Camellia* species, of which 303 were reported from *C. sinensis* according to the U.S. Department of Agriculture (USDA) database [3,4]. Various fungi are known to cause diseases of leaves, stems, and roots of *C. sinensis* and other *Camellia* species, for example, brown blight caused by *Colletotrichum* species, gray blight by *Pestalotiopsis*-like taxa, leaf spots and dieback by *Diaporthe* species, and blister blight by *Exobasidium vexans* [3,32,33]. Our research group studies various foliar diseases allied with tea farms in Taiwan [32,33]. Our previous work has indicated that in addition to the major fungal diseases, such as brown blight due to *Colletotrichum* species complex and grey blight disease caused by *Pestalotiopsis*-like taxa, numerous other fungal species can potentially cause leaf spots on *C. sinensis* in Taiwan [32,33]. Therefore, the aim of this study was to fully characterize the diversity and phylogeny of *Diaporthe*-like strains originally isolated from the leaf spots of *C. sinensis* and evaluate their pathogenicity to the major tea cultivar Chin-Shin Oolong grown in Taiwan’s tea fields. This study was conducted to obtain a better understanding of pathogen biology and determine the optimal temperature and pH for the mycelial growth of these fungi under laboratory conditions.

## 2. Results

### 2.1. Fungal Isolation

**Figure 1:**
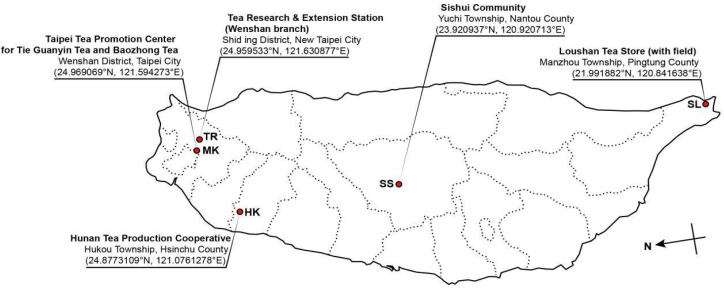
Tea fields surveyed in this study. Relevant geographical details are given for each indicated location.

In total, 19 *Diaporthe*-like strains associated with leaf spots of *C. sinensis* were isolated from five different tea fields located in Taiwan (Figure 1 and Table S1).

### 2.2. Phylogenetic Evaluation of the Concatenated Data Matrix of Diaporthe Species

**Figure 2:**
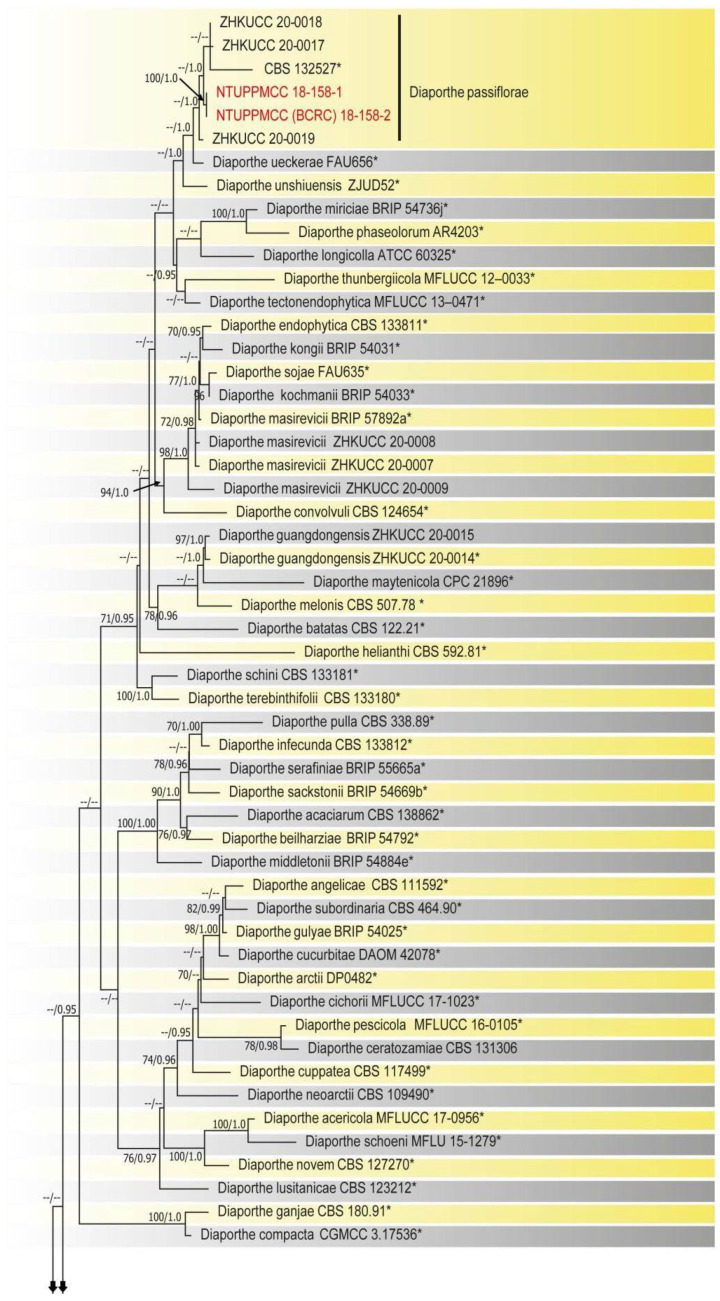

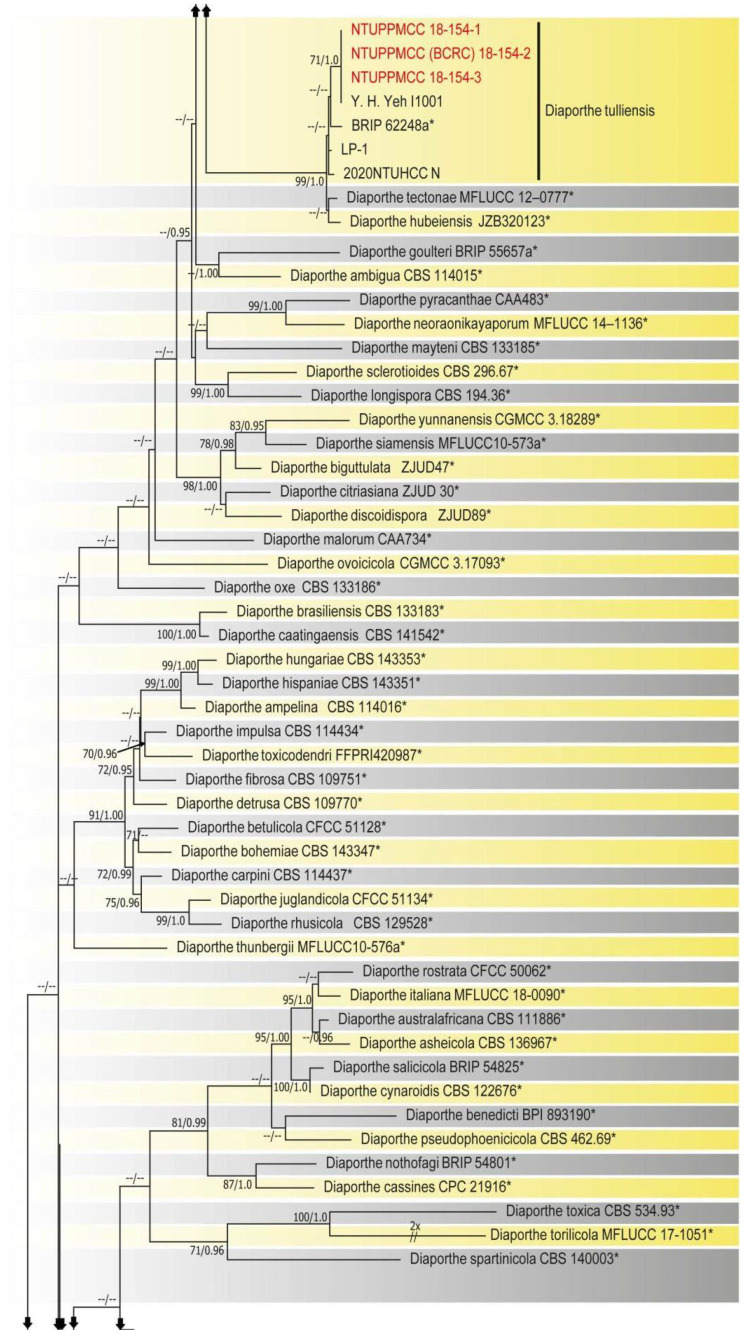

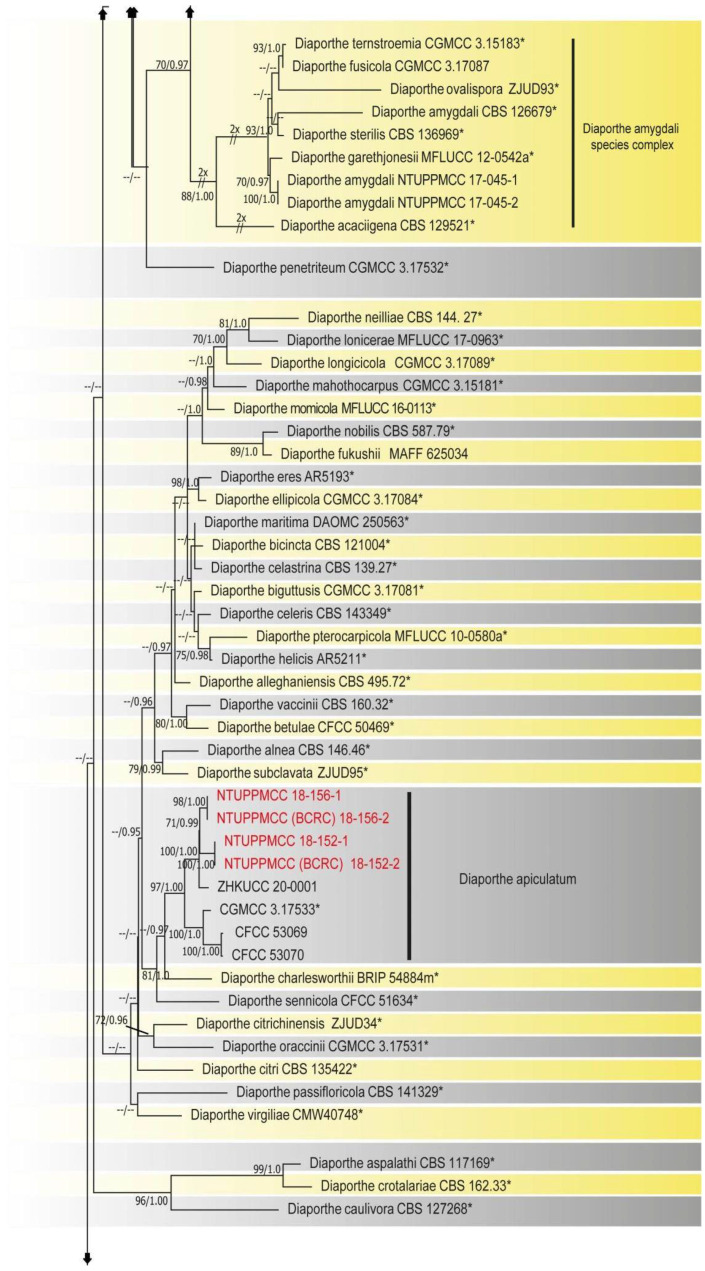

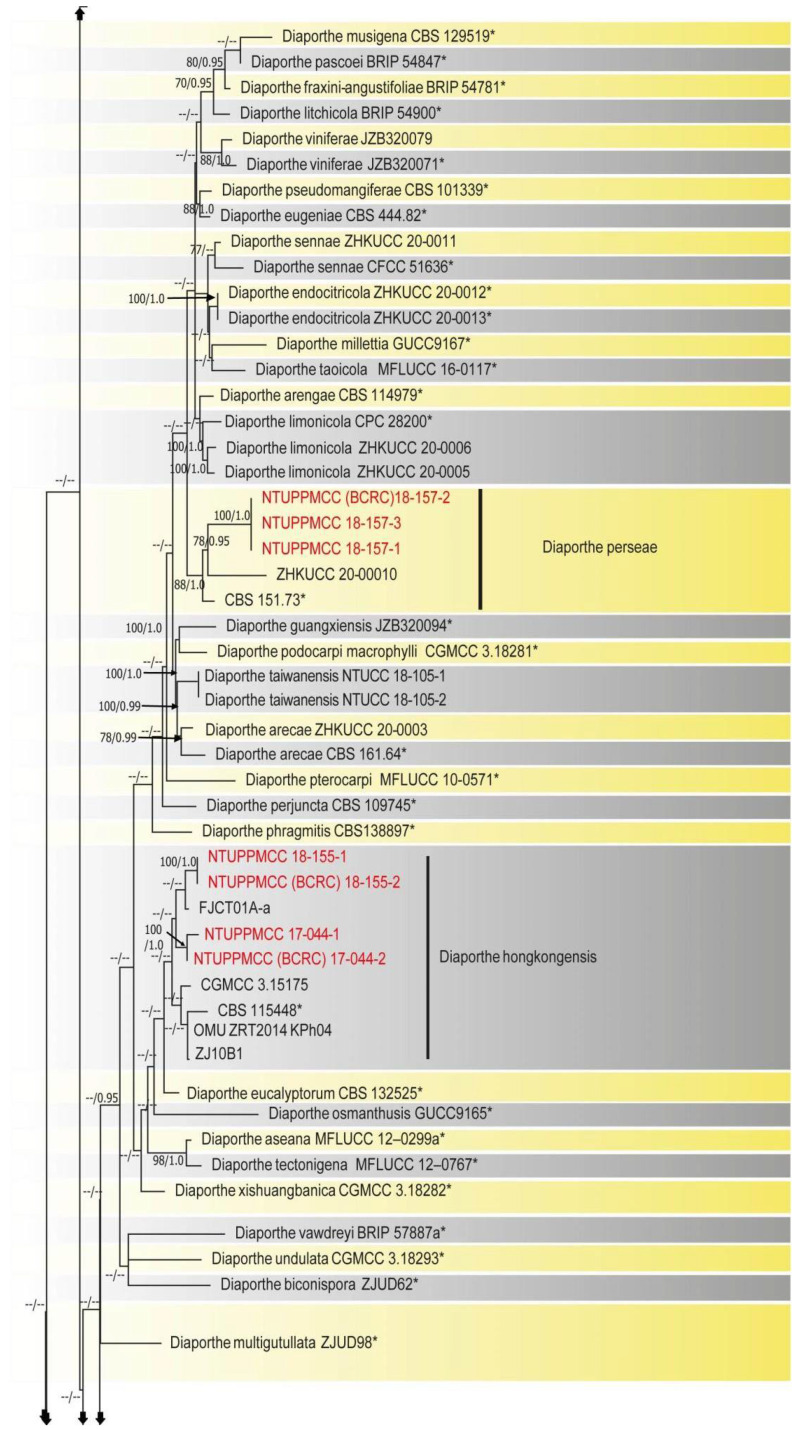

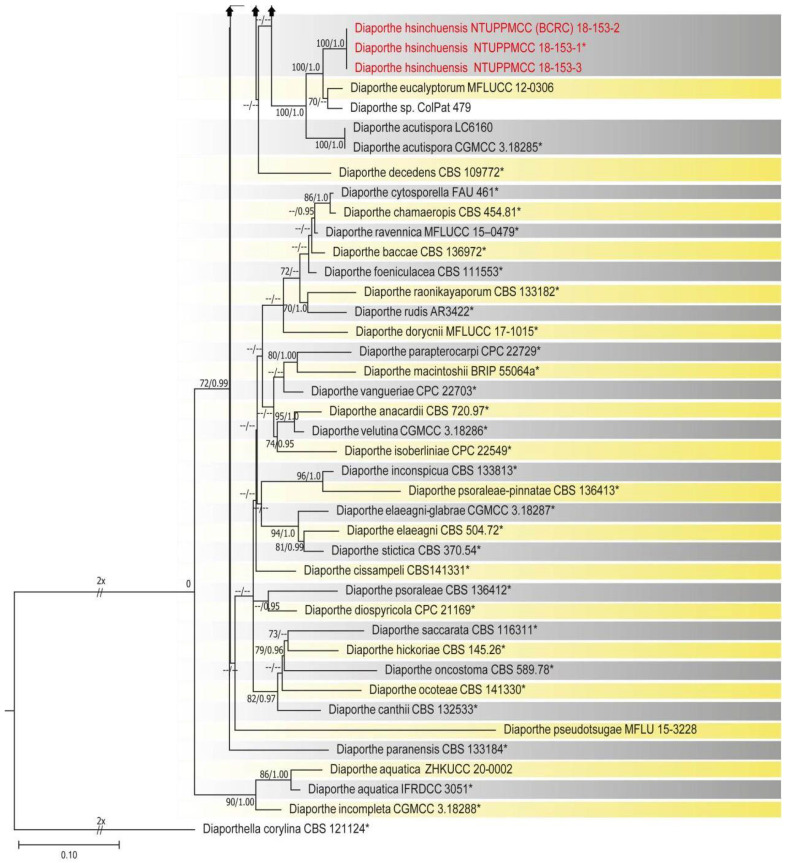
Phylogenetic tree of *Diaporthe* generated from a combined sequence dataset of ITS, *tef1-α*, *tub*2, and *cal* gene regions. Bootstrap values greater than 70% and Bayesian posterior probabilities greater than 0.95 were given below or above the nodes. Isolates obtained in this study are indicated in red, and the ex-type sequences are indicated by *. The strain codes were annotated after relevant species names. *Diaporthella corylina* CBS 121124 served as an outgroup taxon.

Prior to the multilocus gene analysis, alignments corresponding to the gene regions of ITS, *tef1-α*, *tub*2, and *cal* were analyzed using ML. Congruence between single loci made it conceivable to evaluate the phylogenies by concatenating the genes as it delivered a guarantee of gene orthology. The phylogenetic tree obtained from the concatenated gene datasets is in Figure 2.

The final phylogenetic tree was inferred using the combined gene data matrix of ITS, *tef1-α*, *tub*2, and *cal* gene regions from 244 strains of *Diaporthe* species and *Diaporthella corylina* CBS 121124 as the outgroup taxon. The final combined data matrix contained 1803 characters, including gaps (624 for ITS, 526 for *cal*, 381 for *tub*2, and 272 for *tef1-α*). The following priors were used in MrBayes for the five gene loci data partitions based on the results of MrModeltest: all partitions had Dirichlet base frequencies and GTR + I + G models with inverse gamma-distributed rates implemented for ITS and *tub*2, and HKY + I + G with inverse gamma-distributed rates for *cal* and *tef1-α*. The Bayesian analysis resulted in 90,000 trees after 90,000,000 generations. The first 20% of trees representing the burn-in phase of the analyses were discarded, while the remaining trees were used to calculate Bayesian posterior probabilities (PP) in the majority rule consensus tree. The best scoring RAxML tree had a likelihood value of: −4812.054120 and GTR + I + G was used as the evolutionary model.

The phylogenetic trees obtained from both ML and Bayesian analyses had a similar topology and branching pattern based on phenotypic characters and were supported by the molecular phylogenetic inference of strains generated in this study. The results were consistent with recent publications [3,12,13,30,34,35,36]. In the present phylogeny (Figure 2), out of 19 strains, 16 were assigned to previously described species, namely, *D. apiculatum* (4), *D. hongkongensis* (4), *D. tulliensis* (3), *D. passiflorae* (2), and *D. perseae* (3). Three strains in this study formed distinct clades with a highly supported sub-clade, which was identified as a novel species and named *D. hsinchuensis*.

### 2.3. Taxonomy Diaporthe hsinchuensis Ariyawansa and I. Tsai, sp. nov.

**Figure 3:**
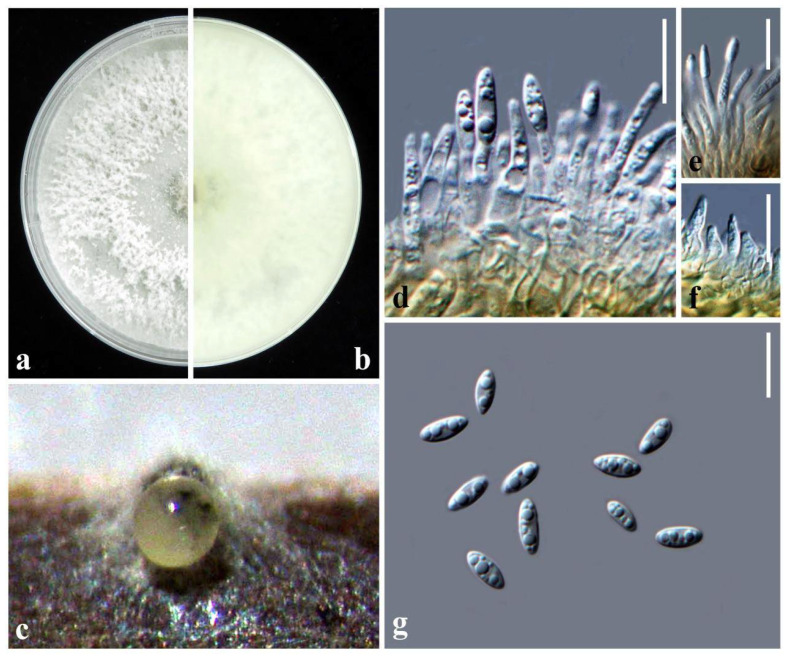
*Diaporthehsinchuensis* NTUPPMCC 18-153-1: (**a**,**b**) surface and reverse side of colony on PDA; (**c**) conidiomata on PNA. (**d**–**f**); conidiogenous cells; (**g**) conidia. Scale bars = 10 µm.

MycoBank: MB840098, Figure 3

*Etymology*: The epithet refers to Hsinchu, Taiwan, where this species was originally collected.

*Description:* Forms grey lesions on the tip of the tea (*Camellia sinensis*) leaf. *Sexual morph*: Undetermined. *Asexual morph*: *Conidiomata* pycnidial on PNA, globose, erumpent when mature, conidia exuding from the pycnidia in ivory white drops. *Conidiophores* cylindrical, phialidic, septate, and branched, 8–20 × 1–3 µm ($\bar{x}$ ± SD = 14.1 ± 2.9 × 2.0 ± 0.4 μm, n = 30). *Alpha conidiogenous cells* hyaline, ovoid to ampulliform, cylindrical to subcylindrical tapering towards the apex, straight or curved, 1–8 × 1–4 μm ($\bar{x}$ ± SD = 5.6 ± 1.9 × 2.4 ± 0.6 μm, n = 30). *Alpha conidia* hyaline, oval, or fusiform, unicellular, aseptate, 2–7-guttulate 6–9 × 2.5–4 μm ($\bar{x}$ ± SD = 7.5 ± 0.9 × 3.2 ± 0.2 μm, n = 30). *Beta conidia* not observed. *Gamma conidia* not observed.

*Colony characteristics*: Colonies on PDA circular, edge entire, surface white, cottony, reverse yellowish white.

*Material examination*: Taiwan, Hsinchu County, Hukou Township, Hunan Tea Production Cooperative, on leaves of *Camellia sinensis* (Theaceae), 4 April 2018, Tsai Ichen, HK04-1 (NTUPPMH 18-153-1, holotype), ex-type culture NTUPPMCC 18-153-1; *ibid*., HK04-2 (NTUPPMH 18-153-2) = living culture NTUPPMCC 18-153-2.

*Notes:* The strains representing *Diaporthe hsinchuensis* clustered in a well-supported clade (ML/PP = 100/1.0) and formed a distinct linage sister to *D. eucalyptorum* (MFLUCC 12-0306), *D. acutispora* (LC6160 and LC6161), and *Diaporthe* sp. (ColPat479) (Figure 2). Unfortunately, morphological data were not available for *D. eucalyptorum* (MFLUCC 12-0306) or *Diaporthe* sp. (ColPat479). Furthermore, the ex-type strain of *D. eucalyptorum* (CBS 132525), which was used by Crous et al. [37] to introduce the species, appeared as a basal clade to strains containing *D. hongkongensis* in the present study in agreement with previous studies [2,30]. This may indicate that *D. eucalyptorum* (MFLUCC 12-0306), used by Senanayake et al. [38], is a different species, but further studies are required to confirm the taxonomic status of this strain. Based on the available data, we only compared the morphological features of *D. hsinchuensis* with *D. acutispora*. *D. hsinchuensis* differs from *D. acutispora* in its smaller conidiophores (8–20 × 1–3 µm versus 10–34.5 × 2–3 μm), relatively smaller alpha conidia (6–9 × 2.5–4 μm versus 7–10 × 2–3 μm), host (*Camellia sinensis* versus *Coffea* sp.), and geographical location (Taiwan versus China). In addition, *D. hsinchuensis* can be clearly differentiated based on nucleotide differences in ITS, *tef1-α, tub*2, and *cal* loci from its phylogenetically closely related species, *D. acutispora* (ITS 4%; *tef1-α* 14%; *tub*2 5%; *cal* 6%).

### 2.4. Growth Rate

**Figure 4:**
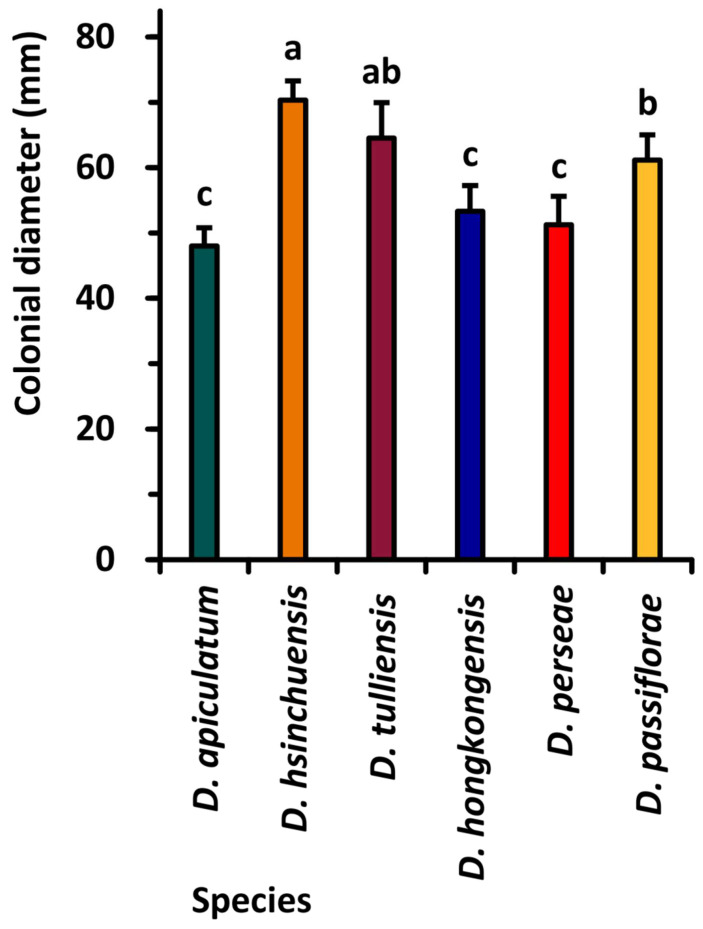
Comparison of mycelial growth rate of the six *Diaporthe* species based on perpendicular colonial diameter. According to Tukey’s range test, data (mean ± standard deviation) with the same letters are not significantly different. Colors identify different taxa.

All tested isolates were grown on PDA media for 7 days at 25 °C in the dark. The sizes of colonies were measured (mm), and their means were calculated and are presented in Figure 4. Isolate NTUPPMCC 18-153-1 (*D. hsinchuensis*) exhibited the widest diameter colony (70.33 mm on average), which, along with isolate NTUPPMCC 18-154-1 (*D. tulliensis*), presented significantly faster growth after 7 days of incubation. In contrast, strain NTUPPMCC 18-152-1 (*D. apiculatum*), 18-155-1 (*D. hongkongensis*), and 18-157-1 (*D. perseae*) had significantly slower growth compared to all the other isolates.

### 2.5. Temperature Effects

**Figure 5:**
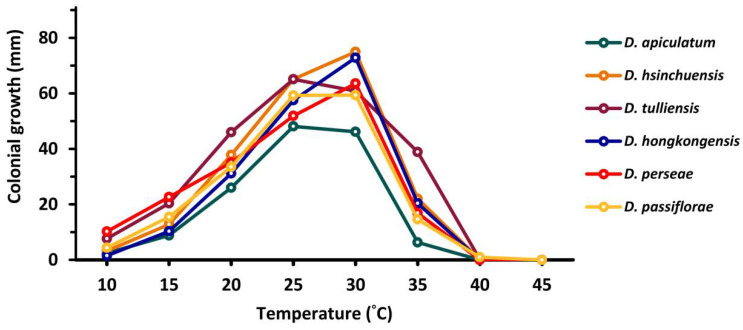
Temperature effect on mycelial growth according to the comparison of colonial growth (mm) of different species at each temperature, based on the mean values presented in Table S2.

Fungal mycelial growth was detected for all the tested isolates between 10 to 45 °C and measured as colony diameter. Temperature regimes strongly influenced the growth of tested fungal strains (*p* ≤ 0.001); the maximum growth was determined at 25–30 °C (mean 60.39 mm), while the minimum or no-growth was observed at 40–45 °C (mean 0.10 mm) for all the isolates (Figure 5, Table S2).

### 2.6. Optimal pH

**Figure 6:**
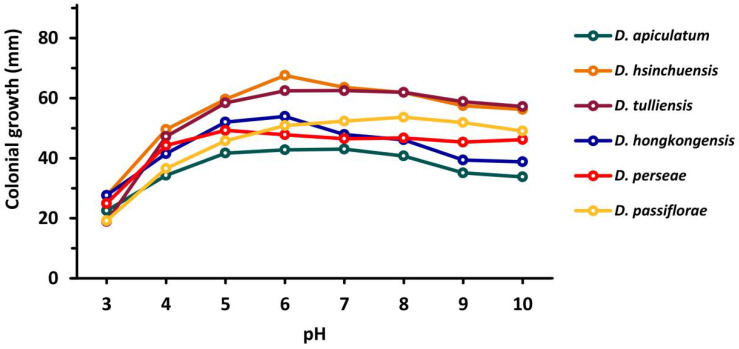
Optimal pH for mycelial growth of each species according to the comparison of colonial growth (mm diam), based on the mean values presented in Table S3.

The effects of pH on the mycelial growth of tested strains are presented in Figure 6 and Table S3. Results showed that, generally, all strains used in this study grow better in slightly acidic to alkaline medium (pH 5–10) compared with acidic medium (pH 3–5). Isolates NTUPPMCC 18-153-1 and 18-155-1 showed a relatively narrower optical pH range (pH 6–8 and 5–7, respectively) upon mycelial growth compared with the other isolates.

### 2.7. Pathogenicity

**Figure 7:**
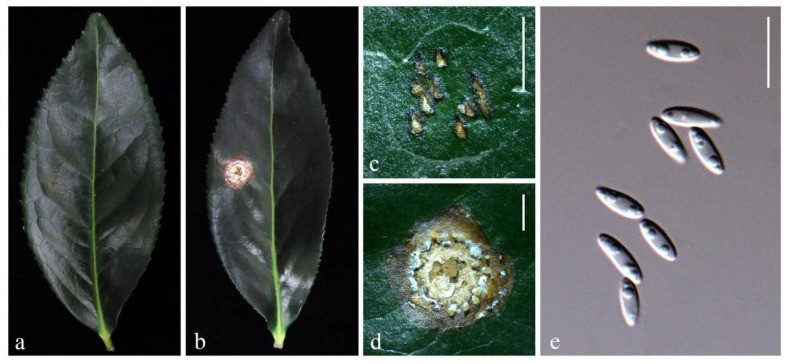
Symptoms caused by *D. hongkongensis*: (**a**,**c**) control leaf inoculated with PDA disk, symptoms absent; (**b**,**d**) symptom on tea leaf inoculated with *D. hongkongensis* 14 days after incubation (lesion size on average = 6.38 mm diam); (**e**) conidia obtained from artificially generated lesion. Scale bars: (**c**,**d**) = 2 mm; (**e**) = 10 μm.

In total, all six selected isolates except NTUPPMCC 18-155-1 (*D. hongkongensis*) failed to cause symptoms on either wounded or unwounded inoculated sites. However, NTUPPMCC 18-155-1 caused symptoms on wounded inoculated sites on tea leaves (Figure 7), showing that the strain might enter tea leaves via wounds. The characteristic of the concentric circular fruiting-body formation and brown blight shared among observed symptoms (Figure 7d) were consistent with that observed in natural tea fields. The fungal strain capable of forming lesions on wounds in this experiment was re-isolated from the fruiting lesions with morphological characteristics identical to those of the original isolates. Therefore, Koch’s postulates were fulfilled and based on both the DNA sequence data and morphological evidence from these re-isolates, all were confirmed to be pathogenic to *C. sinensis*.

## 3. Discussion

Tea is one of the major crops grown in Taiwan, and oolong tea produced by Taiwan is responsible for 25% of global oolong production (One Town One Product (OTOP) [32,33]. Foliar diseases of *C. sinensis* are of concern to tea farmers because leaves are the main part of the plant used to produce various teas. However, research regarding *Diaporthe* species causing foliar diseases of *C. sinensis* is rare, and twig blight caused by unknown *Phomopsis* species (asexual morph of *Diaporthe*) are the only diseases caused by *Diaporthe* mentioned in the list of plant diseases in Taiwan [39].

Accurately naming a fungus based on its molecular and phenotype characters that reflect phylogeny allows predictions about plant-associated organisms together with potential pathogenicity and appropriate control measures. In the present study, we identified six *Diaporthe* taxa linked with leaf spot in Taiwan tea fields, including a novel species. Various *Diaporthe* species have been identified as either pathogens or endophytes on *C. sinensis* such as *D. amygdali*, *D. apiculata*, *D. discoidispora*, *D. eres*, *D. foeniculacea*, *D. foeniculina*, *D. hongkongensis*, *D. incompleta*, *D. longicicola*, *D. masirevicii*, *D. nobilis*, *D. oraccinii*, *D. penetriteum*, *D. portugallica*, *D. tectonigena*, *D. ueckerae*, *D. velutina*, and *D. xishuangbanica* [40]. Among the tested isolates, *D. hongkongensis* fulfilled Koch’s postulates and was identified as the only pathogen causing leaf spot on *C. sinensis* in the present study. *D. hongkongensis* has been recognized as a well-known phytopathogen affecting various hosts such as grapevine [41], peach [42], kiwifruit [43], dragon fruit [44], and pear [15] in recent studies. *D. hongkongensis* was also reported to be associated with both healthy and diseased tea leaves in China by Gao et al. [3]. However, in their study, Gao et al. [3] did not confirm the pathogenicity of *D. hongkongensis* to tea leaves by fulfilling Koch’s postulates. In addition, there is no record of *D. hongkongensis* causing plant diseases in Taiwan. Therefore, to the best of our understanding, this is not only the first report of *D. hongkongensis* causing leaf spot on *C. sinensis*, but also a novel discovery of *D. hongkongensis* present in Taiwan.

Furthermore, in this study, we reported another species associated with tea leaf spot, namely, *D. apiculatum*, for the first time in Taiwan. Gao et al. [3] introduced *D. apiculatum* as a new species from the healthy and infected leaves of *C. sinensis* in China but did not confirm the pathogenicity of the fungal strains via Koch’s postulates. However, in the present study, strains that we identified as *D. apiculatum* did not fulfill Koch’s postulates after 14 days of incubation. Therefore, further studies are required to confirm whether this is a latent pathogen or a conditional pathogen, causing symptoms when the plant defense mechanisms are weak.

In the present study, *Diaporthe passiflorae, D. tulliensis*, and *D. perseae* were isolated from the leaf spots of *C. sinensis* for the first time. *D. passiflorae* was introduced by Crous et al. [37] as a potential endophyte on the fruit of *Passiflora edulis*. In contrast, Li et al. [45] identified the same species as a postharvest pathogen causing fruit rot of Kiwifruit in Sichuan Province, China. *D. perseae* was originally described as *Phomopsis perseae* Zerova from branches of dying *Persea gratissima* trees in Russia [29]. This species also has been identified as a pathogen of mango causing fruit rot in Malaysia [46] and as endophytes on *Citrus grandis* in China [30]. *D. tulliensis* has been recognized as an important pathogen of various hosts, such as kiwifruit [47], coffee [48], and grapes [13]. Interestingly, Huang et al. [49] and Chen and Kirschner [50] recently reported the same species as a pathogen causing leaf spot on *Parthenocissus tricuspidata* as well as an endophyte from *Nelumbo nucifera* in Taiwan, respectively. However, in the present study, none of the strains recognized as *D. passiflorae, D. perseae*, *D. tulliensis*, or the new species *D. hsinchuensis*, satisfied Koch’s postulates after 14 days of incubation. Thus, they were not considered pathogenic to major tea cultivar Ching-Shin Oolong grown in Taiwan.

Only a few studies have shown the effect of environmental factors on the growth of *Diaporthe* species. The most recent study was carried out by Arciuolo et al. [51], who tested the optimal temperature and water activity required for mycelial growth, pycnidial conidiomata development, and asexual spore production of *D. eres*, the causal agent of hazelnut defects. Arciuolo et al. [51] concluded that the optimum temperature for mycelial growth of *D. eres* was observed at 20–25 °C and at 30 °C for pycnidia and cirrhi development. In another study, Hilário et al. [12] observed that four novel pathogens of *Diaporthe*, namely, *D. crousii*, *D. phillipsii*, *D. rossmaniae*, and *D. vacuae*, causing twig blight and dieback of *Vaccinium corymbosum* in Portugal had an optimum temperature for mycelial growth at 20–25 °C. The present study provides insights into the effect of environmental factors such as temperature and pH on the mycelial growth of the *Diaporthe* strains isolated from *C. sinensis* in Taiwan under a controlled environment. Our results showed that all species identified in this study reached maximum colony diameter at 25–30 °C and preferred to grow under low acidic to alkaline rather than acidic medium. The observations in the present study are consistent with the results of Arciuolo et al. [51] and Hilário et al. [12]. In the future, these abiotic factors could be used as the basis for developing a predictive model for the infection of *Diaporthe* taxa of tea plants in Taiwan.

## 4. Conclusions

In conclusion, the research outcomes of the present study improve the understanding of *Diaporthe* species allied with leaf spots on tea leaves and deliver valuable data for effective disease management of *C. sinensis* in Taiwan. The study identified six *Diaporthe* species associated with leaf spots on *C. sinensis* in Taiwan tea fields. These species comprise one novel species and three new records in *Diaporthe* on *C. sinensis*. However, to gain better knowledge of the diversity, pathogenicity, distribution, and implications of *Diaporthe* taxa on tea plantations in Taiwan, extensive surveys on *C. sinensis* plantations and the collection of a larger number of samples should reflect the goals of future studies.

## 5. Materials and Methods

### 5.1. Sample Collection, Fungal Isolation, and Morphological Examination

Tea leaves with characteristic leaf spots were collected from five distinct tea fields located in major tea-growing areas in Taiwan (Figure 1). The samples were collected in resealable plastic bags and carried back to the laboratory. Pure cultures were obtained through a single conidium isolation method, as described in Udayanga et al. [26] and Ariyawansa et al. [4]. In brief, contents of the fruiting body were mounted in a drop of sterile distilled water on a flame-sterilized cavity slide and pipetted to thoroughly mix. The drop of spore suspension was spread evenly on a Petri dish of water agar (WA) and incubated at 25 °C in the dark for 12 h. Single germinating conidia were transferred to a Petri dish of potato dextrose agar (PDA; HiMedia Laboratories Pvt. Ltd., Mumbai, India) and incubated at 25 °C in the dark. Colonial characterization was carried out from isolates cultured on PDA (HiMedia Laboratories Pvt. Ltd., Mumbai, India). Conidiomatal growth was detected on WA with double-autoclaved pine needles placed on the surface of agar (PNA), corn meal agar (CMA; HiMedia Laboratories Pvt. Ltd., Mumbai, India), or PDA supplemented with 10% NaCl, which were incubated at 25 °C under continuous blue light for 7 to 14 days [33]. Microscopic characteristics were examined in distilled water, with 30 measurements taken from each structure using cellSense Standard software (XV Imaging, Version 3.17.0.16686) under an Olympus BX51 microscope (Olympus Corp., Tokyo, Japan) with differential interference contrast (DIC) illumination.

In this study, type specimens were deposited in the herbarium of the Department of Plant Pathology and Microbiology, National Taiwan University (NTUPPMH). Ex-type living cultures were deposited in the Department of Plant Pathology and Microbiology, National Taiwan University Culture Collection (NTUPPMCC), and the Bioresource Collection and Research Centre (BCRC).

### 5.2. DNA Extraction, PCR, and Sequencing

The growing mycelia of each isolate were gathered from cultures incubated at 25 °C in the dark for 7 to 14 days. Genomic DNA were extracted with EasyPure Genomic DNA Spin Kit (Bioman Scientific Co., Ltd., New Taipei, Taiwan) according to the manufacturer’s guidelines (Bioman Scientific Co., Ltd., New Taipei, Taiwan). PCR amplifications of ITS, tef1-α, tub2, and cal gene regions were separately performed in 25 µL reaction mixtures, as described in Ariyawansa et al. [4]. The relevant primer pairs included in this study are in Table 1. The PCR products were visualized by electrophoresis on a 1.5% agarose gel stained with BioGreenTM Safe DNA Gel buffer (Bioman Scientific Co., Ltd., New Taipei, Taiwan). The sequence purified amplicons from each gene region were obtained using the Sanger sequencing method at the Genomics Co., Ltd., (New Taipei, Taiwan). Newly obtained sequences in the present study were deposited to NCBI GenBank.

### 5.3. Strain Selection, Sequence Alignment, and Phylogenetic Analysis

An initial ITS-only tree containing all taxa currently recognized in *Diaporthe* was made to resolve the clades containing the isolates obtained in this study (data not shown). This analysis was further used to select the species to be included in the multilocus phylogenetic analyses. DNA sequence data of ITS, *tef1-α*, *tub*2, and *cal* loci were used to determine the phylogenetic placement of isolates. DNA sequences of each strain were searched against GenBank by nucleotide BLAST (BLASTn) to find the nearest matches. Strains in GenBank with 95–99% similarity to known *Diaporthe* species together with species previously described as pathogens on tea were included in the phylogenetic analysis following the recent publications of Ariyawansa et al. [4], Yang et al. [55], Dissanayake et al. [31,56], Gao et al. [57], Guarnaccia and Crous [8], and Manawasinghe et al. [13]. The strains used in the present study and their GenBank accession numbers are presented in Table S4.

Multiple sequence alignments were generated in MAFFT v. 6.864b with default parameters (http://mafft.cbrc.jp/alignment/server/index.html, accessed on 3 April 2021). The alignments for each gene were visually improved manually where necessary in MEGA v. 5 [58]. Single gene trees were first built for ITS, *tef1-α*, *tub*2, and *cal*, and finally subjected to a multilocus analysis. MrModeltest v. 2.3 [59] under the Akaike Information Criterion (AIC) implemented in PAUP v. 4.0b10 [60] was used to determine the individual selection of evolutionary models for phylogenetic analyses of each locus.

Two phylogenetic tree inference methods, maximum likelihood (ML) in RAxML [61], and Bayesian analyses in MrBayes v. 3.0b4 [62] were used to evaluate the phylogenetic relationships of the strains used in this study, as described in Ariyawansa et al. [4] and Tsai et al. [33]. Bootstrap values obtained via ML (MLB) and Bayesian posterior probabilities (BPP) that were equal to or greater than 70% or greater than 0.95, respectively, are indicated below or above each node (Figure 2). Topologies of the trees obtained from each gene were visually compared to confirm the similarity between the overall tree topology of the individual datasets and that of the tree obtained from the combined alignment. MEGA v. 5 [58] and FigTree v. 1.4 [63] software were used to assess and visualize phylogenetic trees and data files.

The principles of Genealogical Concordance Phylogenetic Species Recognition (GCPSR) were used to identify the species limits within *Diaporthe*-like taxa [64,65]. Dettman et al. [65] proposed that species should be differentiated when fulfilling one of the following two criteria: genealogical concordance or genealogical non-discordance. In other words, if clades exist in at least some of the trees, then they are recognized as genealogically concordant; clades are recognized as genealogically non-discordant if they are significantly supported with high statistical values (MLB ≥ 70, PP ≥ 0.95) in a single locus without conflict at or above this support level in any other single-locus trees. By following this criterion, poorly supported non-monophylies in one locus are eliminated without undermining well-supported monophylies in another locus.

Based on the outcomes of the phylogenetic analysis considering a single strain representing each taxon, a total of six isolates were randomly selected for the pathogenicity, mycelial growth, temperature, and pH tests.

### 5.4. Mycelial Growth Test

In total, six strains representing six taxa identified in this study, NTUPPMCC 18-152-1 (*Diaporthe apiculatum*), NTUPPMCC 18-055-1 (*D. hongkongensis*), NTUPPMCC 18-153-1 (*D. hsinchuensis*), NTUPPMCC 18-154-1 (*D. tulliensis*), NTUPPMCC 18-157-1 (*D. perseae*), and NTUPPMCC 18-158-1 (*D. passiflorae*), were used to determine the radial growth of mycelia. The growth rate was evaluated following a modified procedure of Huang et al. [66]. A 4 mm-diam mycelial disk was cut from the edge of a three-day-old PDA culture, placed centrally into a Petri dish (90 mm diam) of PDA (13 mL) and incubated at 25 °C in the dark for three days. Measurements were taken daily, based on the diameter of two perpendicular axes per fungal colony. The mycelial growth was determined on the final measurement. The test was conducted thrice with three replicates per trial.

### 5.5. Temperature and pH Effects on Mycelial Growth

The same isolates, volume of medium, inoculation method, and standard of measurement used in the mycelial growth test were used to test the effects of temperature and pH. Further details for each assessment are described below.

The effect of temperature on radial mycelial growth was checked on a daily basis and determined on the third day after inoculation at 10, 15, 20, 25, 30, 35, 40, and 45 °C in the dark. All single inoculations were conducted on Petri dishes of PDA. The test was performed three times, with three replicates per trial. The present protocol was modified from Keith et al. [67].

The optimal pH for radial mycelial growth was studied at pH 3, 4, 5, 6, 7, 8, 9, and 10. PDA plates were heated to mix prior to sterilization, and the pH values were adjusted with 1 M HCl and 1 M NaOH solutions [68,69]. The tested cultures were incubated at 25 °C in the dark for three days, and the colony sizes were measured on a daily basis. The test was conducted three times, with three replicates per trial.

### 5.6. Pathogenicity Assessment

The same isolates used in the above temperature and pH investigations were used in the pathogenicity assessment. The test was conducted on detached tea leaves (picked from fourth to sixth leaf below the apical bud) randomly collected from healthy branches of Ching-Shin Oolong gathered from a conventional tea field (ca. 15 years old) located in Pinglin District, New Taipei City (24°56′26.9″ N, 121°43′25.4″ E), as described in Tsai et al. [33]. In brief, fresh leaf-attached tea branches were thoroughly rinsed with tap water to remove dust, and excessive water was gently eliminated with tissue paper. A flat rack (164 × 114 × 26 mm^3^) wrapped with sterile tissue paper was placed in a plastic box (320 × 240 × 70 mm^3^), and the box was filled with 700 mL of sterilized distilled water. Four tea leaves were detached from the branch, surface sterilized with 75% ethanol and fixed to the rack at the foliar tip and base with rubber bands. The leaves were wounded by pinpricking with a sterile needle. A 4 mm-diam mycelial disk was cut from the margin of a seven-day-old colony on PDA and inoculated on a single wounded site [70]. In total, four tea leaves inoculated with PDA disks (4 mm diam) without mycelium served as controls. The box with the above contents was sealed with plastic wrap to maintain moisture and incubated at 26 ± 1 °C under a 12/12 h photocycle [33]. The pathogenicity was determined after 14 days of incubation. The test was conducted three times, with four replicates for each round per isolate.

### 5.7. Statistical Analysis

The statistical analysis was carried out by one-way analysis of variance (ANOVA) using SAS^®^ University Edition v. 3.8, and the pairwise comparison was performed via Tukey’s range test (α = 0.05).

## Figures and Tables

**Table 1:** Gene regions and primer sequences used in this study.

Region	Primers	Sequence (5′→3′)	Reference
ITS	ITS5 ITS4	GGA AGT AAA AGT CGT AAC AAG G TCC GCT TAT TGA TAT GC	[52]
tef1-α	EF1-728F EF1-986R	CAT CGA GAA GTT CGA GAA GG TAC TTG AAG GAA CCC TTA CC	[53]
tub2	Bt2a Bt2b	GGT AAC CAA ATC GGT GCT TTC ACC CTC AGT GTA GTG ACC CTT GGC	[54]
cal	CAL-228F CAL-737R	GAG TTC AAG GAG GCC TTC TCC C CAT CTT TCT GGC CAT GG	[53]

## Data Availability

Publicly available datasets were analyzed in this study. This data can be found here: https://www.ncbi.nlm.nih.gov/ (accessed on 13 July 2021) and https://www.mycobank.org/page/Simple%20names%20search (accessed on 13 July 2021).

## Supplementary Materials

The following are available online at https://www.mdpi.com/article/10.3390/plants10071434/s1, Table S1: collection information of *Diaporthe* taxa used in this study, Table S2: mycelial growth (mm diam., with the origin diameter of inoculated mycelial plug deducted) of each isolate (NTUPPMCC) at every pH level. Data (mean ± standard deviation) with the same letters are not significantly different on the basis of Tukey’s range test. Table S3: GenBank accession numbers of isolates included in the multilocus sequence analysis, Table S4: mycelial growth (mm diam., with the origin diameter of inoculated mycelial plug deducted) of individual isolate (NTUPPMCC) at each temperature. Data (mean ± standard deviation) with the same letters are not significantly different on the basis of Tukey’s range test.
