# Supplementary material for: Molecular Phylogenetic Diversity and Biological Characterization of Diaporthe Species Associated with Leaf Spots of Camellia sinensis in Taiwan
Source: Plants (Basel). 2021 Jul 14;10(7):1434. doi: 10.3390/plants10071434 (PMC8309328; doi:10.3390/plants10071434)
Supplement: Supplementary file 1 [file plants-10-01434-s001.zip › plants-1295910-supplementary.pdf]

Table S1. Collection information of *Diaporthe* taxa used in this study.

Date	Association/field; geographical location	Taxon	Culture No.[a]
23 Nov. 2017	Tea Research and Extension Station (Wenshan branch); Shiding, New Taipei	*D. hongkongensis*	17-044 -1, -2
10 Mar. 2018	Taipei Tea Promotion Center for Tie Guanyin Tea and Baozhong Tea; Wenshan, Taipei	*D. apiculatum*	18-152 -1, -2
04 Apr. 2018	Hunan Tea Production Cooperative; Hukou, Hsinchu	*D. hsinchuensis*; *D. tulliensis*	18-153 -1, -2, -3; 18-154 -1, -2, -3
14 Apr. 2018	Tea fields by Sishui Community Development Association; Yuchi, Nantou	*D. hongkongensis*; *D. apiculatum*	18-155 -1, -2; 18-156 -1, -2
26 May 2018	Laoshan Tea Store (with fields); Manzhou, Pingtung	*D. perseae*; *D. passiflorae*	18-157 -1, -2, -3; 18-158 -1, -2

Notes: [a] NTUPPMCC number.

Table S2. Mycelial growth (mm diam., with the origin diameter of inoculated mycelial plug deducted) of individual isolate (NTUPPMCC) at each temperature. Data (mean ± standard deviation) with the same letters are not significantly different on the basis of Tukey's range test.

T (°C)	Isolates (NTUPPMCC) Colonial growth ± SD (mm)											
	**18-152-1**		**18-153-1**		**18-154-1**		**18-155-1**		**18-157-1**		**18-158-1**	
**10**	2.4 ± 2.54	d	3.2 ± 1.47	f	7.7 ± 2.20	e	1.5 ± 1.15	f	10.2 ± 1.06	e	4.4 ± 1.76	d
**15**	8.8 ± 3.98	c	12.8 ± 3.34	e	20.4 ± 2.27	d	10.3 ± 3.13	e	22.6 ± 2.64	d	15.4 ± 1.61	c
**20**	26.0 ± 1.88	b	37.8 ± 2.37	c	46.0 ± 3.71	b	31.1 ± 4.66	c	35.1 ± 1.47	c	33.5 ± 1.82	b
**25**	48.1 ± 4.76	a	65.1 ± 2.94	b	65.1 ± 4.48	a	57.5 ± 1.55	b	51.9 ± 3.31	b	59.3 ± 1.48	a
**30**	46.2 ± 7.33	a	74.9 ± 2.43	a	60.8 ± 3.13	a	72.8 ± 3.73	a	63.6 ± 4.74	a	59.4 ± 5.46	a
**35**	6.3 ± 2.57	cd	22.1 ± 10.31	d	38.8 ± 9.75	c	20.4 ± 8.00	d	16.9 ± 11.45	de	14.6 ± 6.50	c
**40**	0.0 ± 0.00	e	0.1 ± 0.18	f	0.1 ± 0.18	f	0.0 ± 0.00	f	0.0 ± 0.00	f	1.0 ± 0.71	d
**45**	0.0 ± 0.00	e	0.0 ± 0.00	f	0.0 ± 0.00	f	0.0 ± 0.00	f	0.0 ± 0.00	f	0.0 ± 0.00	d

Notes: Species and respective culture number in this assessment: *D. apiculatum* (NTUPPMCC 18-152-1), *D. hsinchuensis* (NTUPPMCC 18-153-1), *D. tulliensis* (NTUPPMCC 18-154-1), *D. hongkongensis* (NTUPPMCC 18-155-1), *D. perseae* (NTUPPMCC 18-157-1), and *D. passiflorae* (NTUPPMCC 18-158-1).

Table S3. Mycelial growth (mm diam., with the origin diameter of inoculated mycelial plug deducted) of each isolate (NTUPPMCC) at every pH level. Data (mean ± standard deviation) with the same letters are not significantly different on the basis of Tukey's range test.

pH	Isolates (NTUPPMCC) Colonial growth ± SD (mm)											
	**18-152-1**		**18-153-1**		**18-154-1**		**18-155-1**		**18-157-1**		**18-158-1**	
**3**	22.5 ± 1.11	d	27.5 ± 2.25	e	18.9 ± 3.18	c	27.6 ± 2.93	e	24.9 ± 2.15	c	19.2 ± 7.43	c

**4**	34.3 ± 1.89	c	49.5 ± 2.71	d	47.2 ± 3.66	b	41.5 ± 3.81	cd	44.2 ± 4.71	b	36.6 ± 11.69	b
**5**	41.7 ± 7.12	ab	59.6 ± 7.96	bc	58.4 ± 6.33	a	52.0 ± 3.56	ab	49.3 ± 4.19	a	45.8 ± 9.87	ab
**6**	42.8 ± 4.63	a	67.5 ± 4.32	a	62.4 ± 7.67	a	53.9 ± 3.68	a	47.8 ± 3.58	ab	50.8 ± 9.12	ab
**7**	43.0 ± 5.16	a	63.6 ± 2.75	ab	62.5 ± 3.90	a	47.9 ± 5.78	abc	46.5 ± 3.45	ab	52.3 ± 11.42	ab
**8**	40.7 ± 5.68	abc	61.9 ± 3.01	abc	61.9 ± 3.72	a	46.1 ± 9.14	bcd	46.7 ± 3.16	ab	53.6 ± 9.34	a
**9**	35.1 ± 5.82	bc	57.5 ± 2.15	c	58.8 ± 3.16	a	39.4 ± 4.50	d	45.4 ± 1.75	ab	51.8 ± 12.29	ab
**10**	33.8 ± 5.10	c	56.3 ± 4.04	c	57.2 ± 4.56	a	38.8 ± 4.06	d	46.2 ± 1.35	ab	49.1 ± 14.78	ab

Notes: Species and respective culture number in this assessment: *D. apiculatum* (NTUPPMCC 18-152-1), *D. hsinchuensis* (NTUPPMCC 18-153-1), *D. tulliensis* (NTUPPMCC 18-154-1), *D. hongkongensis* (NTUPPMCC 18-155-1), *D. perseae* (NTUPPMCC 18-157-1), and *D. passiflorae* (NTUPPMCC 18-158-1).

Table S4. GenBank accession numbers of isolates included in the multilocus sequence analysis.

**Species**	**Isolate**	**GenBank accession number**			
		ITS	*tub2*	*tef1-α*	*cal*
D. acaciigena	CBS 129521*	KC343005	KC343973	KC343731	KC343247
D. acericola	MFLUCC 17-0956*	KY964224	KY964074	KY964180	KY964137
D. acutispora	CGMCC 3.18285*	KX986764	KX999195	KX999155	KX999274
D. acutispora	LC6160	KX986763	KX999194	KX999154	KX999273
D. alleghaniensis	CBS 495.72*	KC343007	KC343975	KC343733	KC343249
D. alnea	CBS 146.46*	KC343008	KC343976	KC343734	KC343250
D. ambigua	CBS 114015*	KC343010	KC343978	KC343736	KC343252
D. ampelina	CBS 114016*	AF230751	JX275452	AY745056	JX197443
D. amygdali	CBS 126679*	KC343022	KC343990	AY343748	KC343264
D. amygdali	NTUPPMCC 17-045-1	MZ268414	MZ268435	MZ268477	MZ268456
D. amygdali	NTUPPMCC 17-045-2	MZ268415	MZ268436	MZ268478	MZ268457
D. anacardii	CBS 720.97*	KC343024	KC343992	KC343750	KC343266
D. angelicae	CBS 111592*	KC343027	KC343995	KC343753	KC343269
D. apiculata	CGMCC 3.17533*	KP267896	KP293476	KP267970	N/A
D. apiculata	ZHKUCC 20-0001	MT355671	MT409279	MT409325	N/A
D. apiculatum	CFCC 53070	MK432653	MK578056	MK578129	MK442975
D. apiculatum	CFCC 53069	MK432652	MK578055	MK578128	MK442974
D. apiculatum	NTUPPMCC 18-156-1	MZ268416	MZ268437	MZ268479	MZ268458
D. apiculatum	NTUPPMCC 18-156-2	MZ268417	MZ268438	MZ268480	MZ268459
D. apiculatum	NTUPPMCC 18-152-1	MZ268418	MZ268439	MZ268481	MZ268460
D. apiculatum	NTUPPMCC 18-152-2	MZ268419	MZ268440	MZ268482	MZ268461

D. aquatica	IFRDCC 3051*	JQ797437	N/A	N/A	N/A
D. aquatica	ZHKUCC 20-0002	MT355672	MT409280	MT409326	MT409303
D. arctii	DP0482*	KJ590736	KJ610891	KJ590776	KJ612133
D. arecae	CBS 161.64*	KC343032	KC344000	KC343758	KC343274
D. arecae	ZHKUCC 20-0003	MT355673	MT409281	MT409327	MT409304
D. arengae	CBS 114979*	KC343034	KC344002	KC343760	KC343276
D. aseana	MFLUCC 12–0299a*	KT459414	KT459432	KT459448	KT459464
D. asheicola	CBS 136967*	KJ160562	KJ160518	KJ160594	KJ160542
D. aspalathi	CBS 117169*	KC343036	KC344004	KC343762	KC343278
D. australafricana	CBS 111886*	KC343038	KC344006	KC343764	KC343280
D. baccae	CBS 136972*	KJ160565	N/A	KJ160597	N/A
D. batatas	CBS 122.21*	KC343040	KC344008	KC343766	KC343282
D. beilharziae	BRIP 54792*	JX862529	KF170921	JX862535	N/A
D. benedicti	BPI 893190*	KM669929	N/A	KM669785	KM669862
D. betulae	CFCC 50469*	KT732950	KT733020	KT733016	KT732997
D. betulicola	CFCC 51128*	KX024653	KX024657	KX024655	KX024659
D. bicincta	CBS 121004*	KC343134	KC344102	KC343860	KC343376
D. biconispora	ZJUD62*	KJ490597	KJ490418	KJ490476	MT898460
D. biguttulata	ZJUD47*	KJ490582	KJ490403	KJ490461	N/A
D. biguttusis	CGMCC 3.17081*	KF576282	KF576306	KF576257	N/A
D. bohemiae	CBS 143347*	MG281015	MG281188	MG281536	MG281710
D. brasiliensis	CBS 133183*	KC343042	KC344010	KC343768	KC343284
D. caatingaensis	CBS 141542*	KY085927	KY115600	KY115603	N/A
D. canthii	CBS 132533*	JX069864	KC843230	KC843120	KC843174
D. carpini	CBS 114437*	KC343044	KC344012	KC343770	KC343286
D. cassines	CPC 21916*	KF777155	N/A	KF777244	N/A
D. caulivora	CBS 127268*	KC343045	KC344013	KC343771	KC343287
D. celastrina	CBS 139.27*	KC343047	KC344015	KC343773	KC343289
D. celeris	CBS 143349*	MG281017	MG281190	MG281538	MG281712
D. ceratozamiae	CBS 131306	JQ044420	N/A	N/A	N/A
D. chamaeropis	CBS 454.81*	KC343048	KC344016	KC343774	KC343290
D. charlesworthii	BRIP 54884m*	KJ197288	KJ197268	KJ197250	N/A
D. cichorii	MFLUCC 17-1023*	KY964220	KY964104	KY964176	KY964133
D. cissampeli	CBS141331*	KX228273	KX228384	N/A	N/A

D. citri	CBS 135422*	KC843311	KC843187	KC843071	KC843157
D. citriasiana	ZJUD 30*	JQ954645	KC357459	JQ954663	KC357491
D. citrichinensis	ZJUD34*	JQ954648	KJ490396	JQ954666	KC357494
D. compacta	CGMCC 3.17536*	KP267854	KP293434	KP267928	N/A
D. convolvuli	CBS 124654*	KC343054	KC344022	KC343780	KC343296
D. crotalariae	CBS 162.33*	KC343056	KC344024	KC343782	KC343298
D. cucurbitae	DAOM42078*	KM453210	KP118848	KM453211	N/A
D. cuppatea	CBS 117499*	KC343057	KC344025	KC343783	KC343299
D. cynaroidis	CBS 122676*	KC343058	KC344026	KC343784	KC343300
D. cytosporella	FAU461*	KC843307	KC843221	KC843116	KC843141
D. decedens	CBS 109772*	KC343059	KC344027	KC343785	KC343301
D. detrusa	CBS 109770*	KC343061	KC344029	KC343787	KC343303
D. diospyricola	CPC 21169*	KF777156	N/A	N/A	N/A
D. discoidispora	ZJUD89*	KJ490624	KJ490445	KJ490503	N/A
D. dorycnii	MFLUCC 17-1015*	KY964215	KY964099	KY964171	N/A
D. elaegni	CBS 504.72*	KC343064	KC344032	KC343790	KC343306
D. elaegni-glabrae	CGMCC 3.18287*	KX986779	KX999212	KX999171	KX999281
D. ellipicola	CGMCC 3.17084*	KF576270	KF576291	KF576245	N/A
D. endocitricola	ZHKUCC 20-0013	MT355683	MT409291	MT409337	MT409313
D. endophytica	CBS 133811*	KC343065	KC344033	KC343791	KC343307
D. eres	AR5193*	KJ210529	KJ420799	KJ210550	KJ434999
D. eucalyptorum	CBS 132525*	NR120157	N/A	N/A	N/A
D. eucalyptorum	MFLUCC 12-0306	KT459419	KT459437	KT459453	N/A
D. eugeniae	CBS 444.82*	KC343098	KC344066	KC343824	N/A
D. fibrosa	CBS 109751*	KC343099	KC344067	KC343825	KC343341
D. foeniculina	CBS 111553*	KC343101	KC344069	KC343827	KC343343
D. fraxini angustifoliae	BRIP 54781*	JX862528	KF170920	JX862534	N/A
D. fukushii	MAFF 625034	JQ807469	N/A	JQ807418	N/A
D. fusicola	CGMCC 3.17087*	KF576281	KF576305	KF576256	KF576233
D. ganjae	CBS 180.91*	KC343112	KC344080	KC343838	KC343354
D. garethjonesii	MFLUCC 12-0542a*	KT459423	KT459441	KT459457	KT459470
D. goulteri	BRIP 55657a*	KJ197290	KJ197270	KJ197252	N/A
D. guangdongensis	ZHKUCC 20-0014*	MT355684	MT409292	MT409338	MT409314
D. guangdongensis	ZHKUCC 20-0015	MT355685	MT409293	MT409339	MT409315

D. guangxiensis	JZB320094*	MK335772	MK500168	MK523566	MK736727
D. gulyae	BRIP 54025*	JF431299	N/A	JN645803	N/A
D. helianthi	CBS 592.81*	KC343115	KC344083	KC343841	KC343357
D. helicis	AR5211*	KJ210538	KJ420828	KJ210559	KJ435043
D. hickoriae	CBS 145.26*	KC343118	KC344086	KC343844	KC343360
D. hispaniae	CBS 143351 *	MG281123	MG281296	MG281644	MG281820
D. hongkongensis	CBS 115448*	KC343119	KC344087	KC343845	KC343361
D. hongkongensis	FJCT01A-a	KY433561	N/A	KY433569	N/A
D. hongkongensis	ZJ10B1	KY433562	N/A	KY433571	N/A
D. hongkongensis	OMU-ZRT2014-KPh04	LT601560	LT601564	LT601568	LT601572
D. hongkongensis	NTUPPMCC 18-155-1	MZ268423	MZ268444	MZ268486	MZ268465
D. hongkongensis	NTUPPMCC 18-155-2	MZ268424	MZ268445	MZ268487	MZ268466
D. hongkongensis	NTUPPMCC 17-044-1	MZ268425	MZ268446	MZ268488	MZ268467
D. hongkongensis	NTUPPMCC 17-044-2	MZ268426	MZ268447	MZ268489	MZ268468
D. hsinchuensis	NTUPPMCC 18-153-1	MZ268427	MZ268448	MZ268490	MZ268469
D. hsinchuensis	NTUPPMCC 18-153-2	MZ268428	MZ268449	MZ268491	MZ268470
D. hsinchuensis	NTUPPMCC 18-153-3	MZ268429	MZ268450	MZ268492	MZ268471
D. hubeiensis	JZB320123*	MK335809	MK500148	MK523570	MK500235
D. hungariae	CBS 143353*	MG281126	MG281299	MG281647	MG281823
D. impulsa	CBS 114434*	KC343121	KC344089	KC343847	KC343363
D. incompleta	CGMCC 3.18288*	KX986794	KX999226	KX999186	KX999289
D. inconspicua	CBS 133813*	KC343123	KC344091	KC343849	KC343365
D. infecunda	CBS 133812*	KC343126	KC344094	KC343852	KC343368
D. isoberliniae	CPC 22549*	KJ869133	KJ869245	N/A	N/A
D. italiana	MFLUCC 18-0090*	MH846237	MH853688	MH853686	MH853690
D. juglandicola	CFCC 51134*	KU985101	KX024634	KX024628	KX024616
D. kochmanii	BRIP 54033*	JF431295	N/A	JN645809	N/A
D. kongii	BRIP 54031*	JF431301	N/A	JN645797	N/A
D. limonicola	CPC 28200*	MF418422	MF418582	MF418501	MF418256
D. limonicola	ZHKUCC 20-0005	MT355675	MT409283	MT409329	MT409306
D. limonicola	ZHKUCC 20-0006	MT355676	MT409284	MT409330	MT409307
D. litchicola	BRIP 54900*	JX862533	KF170925	JX862539	N/A
D. lithocarpus	CGMCC 3.15175*	KC153104	KF576311	KC153095	KF576235
D. longicicola	CGMCC 3.17089*	KF576267	KF576291	KF576242	N/A

D. longicolla	ATCC 60325*	KJ590728	KJ610883	KJ590767	N/A
D. longispora	CBS 194.36*	KC343135	KC344103	KC343861	KC343377
D. lonicerae	MFLUCC 17-0963*	KY964190	KY964073	KY964146	KY964116
D. lusitanicae	CBS 123212*	KC343136	KC344104	KC343862	KC343378
D. macinthoshii	BRIP 55064a*	KJ197289	KJ197269	KJ197251	N/A
D. mahothocarpus	CGMCC 3.15181*	KC153096	KF576312	KC153087	N/A
D. malorum	CAA734*	KY435638	KY435668	KY435627	KY435658
D. maritima	DAOMC 250563*	N/A	KU574616	N/A	N/A
D. masirevicii	BRIP 57892a*	KJ197277	KJ197257	KJ197239	N/A
D. masirevicii	ZHKUCC 20-0007	MT355677	MT409285	MT409331	MT409308
D. masirevicii	ZHKUCC 20-0008	MT355678	MT409286	MT409332	MT409309
D. masirevicii	ZHKUCC 20-0009	MT355679	MT409287	MT409333	MT409310
D. mayteni	CBS 133185*	KC343139	KC344107	KC343865	KC343381
D. maytenicola	CPC 21896*	KF777157	KF777250	N/A	N/A
D. melonis	CBS 507.78 *	KC343141	KC344109	KC343867	KC343383
D. middletonii	BRIP 54884e*	KJ197286	KJ197266	KJ197248	N/A
D. millettia	GUCC9167*	MK398674	MK502089	MK480609	MK502086
D. miriciae	BRIP 54736j*	KJ197282	KJ197262	KJ197244	N/A
D. momicola	MFLUCC 16-0113*	KU557563	KU557587	KU557631	KU557611
D. multigutullata	ZJUD98*	KJ490633	KJ490454	KJ490512	N/A
D. musigena	CBS 129519*	KC343143	KC344111	KC343869	KC343385
D. neilliae	CBS 144. 27*	KC343144	KC344112	KC343870	KC343386
D. neoarctii	CBS 109490*	KC343145	KC344113	KC343871	KC343387
D. neoraonikayaporum	MFLUCC 14–1136*	KU712449	KU743988	KU749369	KU749356
D. nobilis	CBS 587.79*	KC343153	KC344121	KC343879	KC343395
D. nothofagi	BRIP 54801*	JX862530	KF170922	JX862536	N/A
D. novem	CBS 127270*	KC343155	KC344123	KC343881	KC343397
D. ocoteae	CBS 141330*	KX228293	KX228388	N/A	N/A
D. oncostoma	CBS 589.78*	KC343162	KC344130	KC343888	KC343404
D. oraccini	CGMCC 3.17531*	KP267863	KP293443	KP267937	N/A
D. osmanthusis	GUCC9165	MK398675	MK502090	MK480610	MK502087
D. ovalispora	ZJUD93*	KJ490628	KJ490449	KJ490507	N/A
D. ovoicicola	CGMCC 3.17093*	KF576265	KF576289	KF576240	KF576223
D. oxe	CBS 133186*	KC343164	KC344132	KC343890	KC343406

D. paranensis	CBS 133184*	KC343171	KC344139	KC343897	KC343413
D. parapterocarpi	CPC 22729*	KJ869138	KJ869248	N/A	N/A
D. pascoei	BRIP 54847*	JX862532	KF170924	JX862538	N/A
D. passiflorae	CBS 132527*	JX069860	N/A	N/A	N/A
D. passiflorae	ZHKUCC 20-0017	MT355687	MT409295	MT409341	MT409317
D. passiflorae	ZHKUCC 20-0018	MT355688	MT409296	MT409342	MT409318
D. passiflorae	ZHKUCC 20-0019	MT355689	MT409297	MT409343	MT409319
D. passifloricola	CBS 141329*	KX228292	KX228387	N/A	N/A
D. penetriteum	CGMCC 3.17532*	KP267879	KP293459	KP267953	N/A
D. perjuncta	CBS 109745*	KC343172	KC344140	KC343898	KC343414
D. perseae	CBS 151.73*	KC343173	KC344141	KC343899	KC343415
D. perseae	ZHKUCC 20-0010	MT355680	MT409288	MT409334	N/A
D. perseae	NTUPPMCC 18-157-1	MZ268420	MZ268441	MZ268483	MZ268462
D. perseae	NTUPPMCC 18-157-2	MZ268421	MZ268442	MZ268484	MZ268463
D. perseae	NTUPPMCC 18-157-3	MZ268422	MZ268443	MZ268485	MZ268464
D. pescicola	MFLUCC 16-0105*	KU557555	KU557579	KU557623	KU557603
D. phaseolorum	AR4203*	KJ590738	KJ610893	KJ590739	KJ612135
D. phragmitis	CBS 138897*	KP004445	KP004507	N/A	N/A
D. podocarpi macrophylli	CGMCC 3.18281*	KX986774	KX999207	KX999167	KX999278
D. pseudomangiferae	CBS 101339*	KC343181	KC344149	KC343907	KC343423
D. pseudophoenicicola	CBS 462.69*	KC343184	KC344152	KC343910	KC343426
D. pseudotsugae	MFLU 15-3228	KY964225	KY964108	KY964181	KY964138
D. psoraleae	CBS 136412*	KF777158	KF777251	KF777245	N/A
D. psoraleae pinnatae	CBS 136413*	KF777159	KF777252	N/A	N/A
D. pterocarpi	MFLUCC 10-0571*	JQ619899	JX275460	JX275416	JX197451
D. pterocarpicola	MFLUCC 10-0580a*	JQ619887	JX275441	JX275403	JX197433
D. pulla	CBS 338.89*	KC343152	KC344120	KC343878	KC343394
D. pyracanthae	CAA483*	KY435635	KY435666	KY435625	KY435656
D. raonikayaporum	CBS 133182*	KC343188	KC344156	KC343914	KC343430
D. ravennica	MFLUCC 15–0479*	KU900335	KX432254	KX365197	N/A
D. rhusicola	CBS 129528*	JF951146	KC843205	KC843100	KC843124
D. rostrata	CFCC 50062*	KP208847	KP208855	KP208853	KP208849
D. rudis	AR3422*	KC843331	KC843177	KC843090	KC843146
D. rumicicola	MFLU 18-0739*	MH84623	MK049555	MK049554	N/A

D. saccarata	CBS 116311*	KC343190	KC344158	KC343916	KC343432
D. sackstonii	BRIP 54669b*	KJ197287	KJ197267	KJ197249	N/A
D. salicicola	BRIP 54825*	JX862531	JX862531	JX862537	N/A
D. schini	CBS 133181*	KC343191	KC344159	KC343917	KC343433
D. schoeni	MFLU 15-1279*	KY964226	KY964109	KY964182	KY964139
D. sclerotioides	CBS 296.67*	KC343193	KC344161	KC343919	KC343435
D. sennae	CFCC 51636*	KY203724	KY228891	KY228885	KY228875
D. sennae	ZHKUCC 20-0011	MT355681	MT409289	MT409335	MT409311
D. sennicola	CFCC 51634*	KY203722	KY228889	KY228883	KY228873
D. serafiniae	BRIP 55665a*	KJ197274	KJ197254	KJ197236	N/A
D. siamensis	MFLUCC10-573a*	JQ619879	JX275429	JX275393	N/A
D. sojae	FAU635*	KJ590719	KJ610875	KJ590762	KJ612116
D. spartinicola	CBS 140003*	KR611879	N/A	N/A	N/A
D. sterilis	CBS 136969*	KJ160579	KJ160528	KJ160611	KJ160548
D. stictica	CBS 370.54*	KC343212	KC344180	KC343938	KC343454
D. subclavata	ZJUD95*	KJ490630	KJ490451	KJ490509	N/A
D. subordinaria	CBS 464.90*	KC343214	KC344182	KC343940	KC343456
D. taoicola	MFLUCC 16-0117*	KU557567	KU557591	KU557635	N/A
D. tectonae	MFLUCC 12–0777*	KU712430	KU743977	KU749359	KU749345
D. tectonendophytica	MFLUCC 13–0471*	KU712439	KU743986	KU749367	KU749354
D. tectonigena	MFLUCC 12–0767*	KU712429	KU743976	KU749371	KU749358
D. terebinthifolii	CBS 133180*	KC343216	KC344184	KC343942	KC343458
D. ternstroemia	CGMCC 3.15183*	KC153098	N/A	KC153089	N/A
D. thunbergii	MFLUCC10-576a*	JQ619893	JX275449	JX275409	JX197440
D. thunbergiicola	MFLUCC 12–0033*	KP715097	N/A	KP715098	N/A
D. torilicola	MFLUCC 17-1051*	KY964212	KY964096	KY964168	KY964127
D. toxica	CBS 534.93*	KC343220	KC344188	KC343946	KC343462
D. toxicodendri	FFPRI420987*	LC275192	LC275224	LC275216	LC275200
D. tulliensis	BRIP 62248a*	KR936130	KR936132	KR936133	N/A
D. tulliensis	LP-1	KX457967	N/A	KX457964	N/A
D. tulliensis	2020NTUHCC-N	MT974186	N/A	N/A	N/A
D. tulliensis	Y. H. Yeh I1001	MK336513	N/A	N/A	N/A
D. tulliensis	NTUPPMCC 18-154-1	MZ268411	MZ268432	MZ268474	MZ268453
D. tulliensis	NTUPPMCC 18-154-2	MZ268412	MZ268433	MZ268475	MZ268454

D. tulliensis	NTUPPMCC 18-154-3	MZ268413	MZ268434	MZ268476	MZ268455
D. ueckerae	FAU656*	KJ590726	KJ610881	KJ590747	KJ612122
D. undulata	CGMCC 3.18293*	KX986798	KX999230	KX999190	N/A
D. unshiuensis	ZJUD52*	KJ490587	KJ490408	KJ490466	N/A
D. vaccinii	CBS 160.32*	AF317578	KC344196	GQ250326	KC343470
D. vangueriae	CPC 22703*	KJ869137	KJ869247	N/A	N/A
D. vawdreyi	BRIP 57887a*	KR936126	KR936128	KR936129	N/A
D. velutina	CGMCC 3.18286*	KX986790	KX999223	KX999182	N/A
D. viniferae	JZB320071*	MK341551	MK500112	MK500107	MK500119
D. viniferae	JZB320079	MK341556	MK500118	MK500111	MK500125
D. virgiliae	CMW40748*	KP247566	KP247575	N/A	N/A
D. xishuangbanica	CGMCC 3.18282*	KX986783	KX999216	KX999175	N/A
D. yunnanensis	CGMCC 3.18289*	KX986796	KX999228	KX999188	KX999290
D.endocitricola	ZHKUCC 20-0012*	MT355682	MT409290	MT409336	MT409312
D.passiflorae	NTUPPMCC 18-158-1	MZ268409	MZ268430	MZ268472	MZ268451
D.passiflorae	NTUPPMCC 18-158-2	MZ268410	MZ268431	MZ268473	MZ268452
Diaporthe acaciarum	CBS 138862*	KP004460	KP004509	N/A	N/A
Diaporthe sp.	ColPat-479	MN654334	MN839589	MN706181	N/A
Diaporthella corylina	CBS 121124*	KC343004	KC343972	KC343730	KC343246

Notes: The asterisk symbols indicate sequences of ex-type stains. Newly joined strains and respective sequences included in the present study are in red. N/A, not available.
